# Elevated Homocysteine and Cognitive Impairment in Parkinson's Disease: A Systematic Review and Meta‐Analysis

**DOI:** 10.1002/alz70861_108293

**Published:** 2025-12-23

**Authors:** Yu‐Hsien Chang

**Affiliations:** ^1^ China Medical University, Taichung, Taiwan Taiwan

## Abstract

**Background:**

Cognitive impairment (CI) is a prevalent non‐motor manifestation of Parkinson’s disease (PD), substantially impacting quality of life. Elevated homocysteine (Hcy), a neurotoxic amino acid, has been associated with cognitive dysfunction, yet its relationship with CI in PD remains uncertain. This systematic review and meta‐analysis aimed to determine whether elevated peripheral Hcy levels are associated with cognitive impairment in PD patients.

**Method:**

We systematically searched PubMed, Web of Science, CINAHL, and the Cochrane Library from inception to March 25th 2025 for observational studies comparing Hcy levels between cognitively impaired (PD‐CI) and cognitively normal (PD‐NC) individuals with PD. Study selection, data extraction, and risk of bias assessment (AHRQ checklist) were independently conducted by two reviewers. Random‐effects meta‐analysis, subgroup analysis by region, meta‐regression for L‐dopa dosage and disease duration, leave‐one‐out sensitivity analysis, and Egger’s test were performed. This review was registered in PROSPERO (CRD42025643475).

**Result:**

Twelve studies (n = 1,848; 664 PD‐CI, 1,184 PD‐NC) were included. PD‐CI patients had significantly higher Hcy levels than PD‐NC patients (mean difference: 3.11 μmol/L; 95% CI: 2.13 to 4.10; *p* < 0.00001). Subgroup analysis revealed a stronger association in Eastern populations. Meta‐regression showed a positive association with disease duration (p = 0.0154), but not with L‐dopa dosage. Sensitivity analysis confirmed the robustness of results. No major publication bias was detected.

**Conclusion:**

Elevated peripheral Hcy levels are significantly associated with cognitive impairment in PD. Hcy may serve as a potential biomarker for PD‐related cognitive decline, supporting its utility in clinical risk stratification and longitudinal monitoring.

